# Endocrine treatment in advanced prostate cancer: When to start?

**Published:** 2009

**Authors:** A. V. Choudhrie, J. C. Singh

**Affiliations:** Department of Urology, Christian Medical College, Vellore, India. E-mail: chandrasingh@cmcvellore.ac.in

## SUMMARY

In this trial,[1] 234 patients with histologically proven prostate carcinoma (PCa) and nodal metastases (pN1-3) were randomized to immediate (EET) or delayed endocrine treatment (DET). The primary tumor was not treated. Endocrine treatment consisted of either surgical castration or depot luteinizing hormone-releasing hormone (LHRH) agonist and four weeks of an anti-androgen. The primary objective was to show noninferiority of delayed ET to immediate ET. Median follow-up was 13 years. The median protocol treatment duration was 2.7 years in the delayed and 3.2 years in the immediate ET groups. All except three patients were treated as randomized. Of the 193 patients (82.5%) who died (97 on delayed ET and 96 on immediate ET), 59.4% of them were as a result of prostate cancer. Intention-to-treat analysis revealed a 22% increase in the death hazard in those randomized-to-delayed treatment (HR = 1.22, 95% confidence interval [CI]: 0.92, 1.62). The difference was not statistically significant, but noninferiority was also not proved. The median overall on EET was 7.6 years (95% CI, 6.3–8.3 year) *vs* 6.1 years (95% CI, 5.7–7.3 year) in the DET group. The 10-year cumulative incidence of death was 55.6% in the delayed ET group vs 52.1% with immediate ET group. The authors concluded that this study does not show noninferiority of DET to EET in patients with T2-3PN1-3 M0 prostate carcinoma.

## COMMENTS

The timing of endocrine treatment (ET) for prostate cancer (PCa) without local treatment of the primary tumor remains controversial and this issue was addressed in this trial for patients with lymph node-positive (pN1-3) cancer. Endocrine treatment of advanced prostate cancer is recognized as being palliative and is almost invariably used in men with metastasis. Androgen deprivation is associated with side effects. In the asymptomatic men with advanced prostate cancer at the time of diagnosis, adverse effects of endocrine treatment may be the only ‘symptoms’. This underscores the need to achieve a balance between the impact of ET on the general well-being and quality-of-life against the potential survival benefit.

The studies carried out earlier by the Veterans Administration Co-operative Research Group dealt with the issue of EET *vs* DET.[2] The first study included castration and castration plus diethylstilbestrol. During the course of a 9-year follow-up period, virtually all men who progressed with metastasis in the control arm were eventually treated endocrinologically. Since there was no difference in overall survival with respect to the original randomization, one important conclusion of the trials was that ET could be delayed without impairing overall survival. Though there was a positive effect of early treatment on PC-related mortality in these trials, this was completely neutralized by an increase in the cardiovascular-related death in the diethylstilbesterol DES arms. Cancer-specific survival was marginally improved in those arms with immediate treatment in European Organisation for Research and Treatment of Cancer (EORTC) protocol 30846,[3] protocol 06-88 of the Swiss Oncology Group,[4] and the Medical Research Council (MRC) Prostate Cancer trial in 1997.[5] However, this translated into an advantage in overall survival only in the MRC trial. This favorable difference may be at least partly caused by the fact that 29 out of the 54 cases that died in the DET arm in excess had never received ET. In the MRC trial, significant differences were seen in favor of EET with respect to important disease-related complications. But none of the studies measured quality-of-life. It can be concluded from the findings of these trials that though no credible and clinically relevant advantage in overall survival was seen, EET produces a modest improvement in prostate cancer-specific survival. This has to be weighed against the quality-of-life enjoyed in the first 1.8 years after diagnosis in the DET group without ET, after which progression occurs in the DET arm necessitating hormonal treatment.
